# Olive Tree Derivatives and Hydroxytyrosol: Their Potential Effects on Human Health and Its Use as Functional Ingredient in Meat

**DOI:** 10.3390/foods10112611

**Published:** 2021-10-28

**Authors:** Lorena Martínez-Zamora, Rocío Peñalver, Gaspar Ros, Gema Nieto

**Affiliations:** Department of Food Technology, Nutrition and Food Science, Veterinary Faculty, Campus de Espinardo, University of Murcia, 30100 Murcia, Spain; lorena.martinez23@um.es (L.M.-Z.); rocio.penalver@um.es (R.P.); gros@um.es (G.R.)

**Keywords:** *Olea europaea*, functional, antioxidant, antimicrobial, anti-inflammatory, meat, clean label, food

## Abstract

Olive (*Olea europaea*) is one of the most extensive crops in the Mediterranean countries, and an important source of extra distinctive compounds that has been widely tested due to its known health benefits. Olive derivatives, such as extra virgin olive oil (EVOO) and olive leaves are rich in antioxidant compounds such as hydroxytyrosol (HXT) and oleuropein and oleic acid, as main monounsaturated fatty acid. Because of HXT molecular structure, its regular consumption reports important beneficial properties such as anti-inflammatory, antimicrobial, antioxidant, and anticancer. As a matter of fact, its antioxidant and antimicrobial effects made this compound a good preservative agent against meat deterioration and spoilage, capable of replacing some synthetic additives whose continued and regular consumption may negatively affect the human health. On the contrary side, this extract has an unpleasant odor and flavor, so a synthetic source of HXT could also be used to improve the sensory quality of the meat products. In this sense, this review exposes the health benefits provided by the consumption of EVOO and HXT, and the newest research about its application on meat, together new trends about its use as functional ingredient in meat and meat products.

## 1. Introduction

Nowadays, consumer concerns have increased to demand new healthy and safer foods. One reason is the potential risk of the consumption of some synthetic additives such as sulphites, nitrites, BHA, or BHT, which are widely used as preservatives in most of the animal products that are frequently included in the occidental diet [1,2,3,4,5]. Based on this concern on heath perception, there is a new research trend to achieve the reduction and/or substitution of these synthetic compounds by natural extracts or essential oils from fruits, plants, or spices [6,7,8]. Additionally, most of these natural extracts shown to be antioxidants in meat and fish, but they have a negative impact on organoleptic characteristics of foods due to their high concentration in terpenoids and phenolic. For this reason, its commercial application would not be viable, despite being focused on a population increasingly aware of its health and that demands products free of synthetic additives [9].

In this sense, a part of this field of research is to study the different ways to produce, select, and combine natural extracts or essential oils that do not modify sensory characteristics of animal origin products while maintaining their antioxidant, antimicrobial, and preservative potential. Therefore, the main objective of this research field is to achieve a variety of animal origin products free of artificial ingredients by using organic plant and fruits extracts obtained from food industry by-products, specially from traditional Mediterranean ingredients, among others.

Animals have been the principal food source of proteins for humans since 5 million years ago. However, in last century, the excessive intake of animal protein has influenced on human health, since population is more sedentary than before and combined with the high fat food intake, an increment in heart diseases is produced [10]. In addition, meat is an important source of 25 essential and non-essential elements. These compounds are oxygen, carbon, hydrogen, nitrogen, minerals (Fe–heme, Ca, P, K, S, Na, Cl, Zn, Mg, and Se), and vitamins (A, complex B -B1, B2, B3, B6, B9, B12-, D, and K) [11]. In this sense, meat and meat products are an important source of high-quality proteins, and are also necessary for a balanced diet.

In this way, the application of natural ingredients to avoid the meat spoilage and to allow the total or partial substitution of synthetic additives has been highly increased during las twenty years. In fact, as non-edible parts of fruits and vegetables are especially rich in bioactive compounds, these have become important sources of natural extracts for its use in food and pharmaceutical industry.

One of the most antioxidant compounds known in the world comes directly from the main pillar of the Mediterranean diet (MD): the olive oil. In consequence, first analyses of olive by-products started in 1999 [12] and the process for obtaining hydroxytyrosol (HXT) from olive leaves was patented in 2004 by Beverungen, C. (EP1582512A1). From this moment to today, the number of research related to this compound ascend to 21.400. Indeed, in 2018, our research group launched a review describing the nutraceutical activity of this phytochemical and its use in food industry [13].

The objective of this work is to review the latest literature about olive derivatives and HXT consumption benefits, its extraction, its use as natural preservative, and its application in meat and meat products with special emphasis on perspectives and new trends in meat industry. In addition, we will also focus on new research into new synthetic sources of HXT and its application in industry.

## 2. Olive Tree and Derivatives

**Figure 1:**
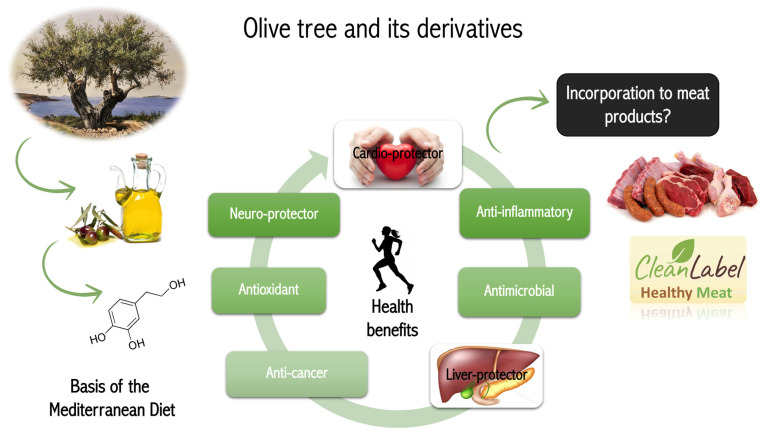
Schematic representation of HXT health benefits and its use as meat preservative.

The olive tree (*Olea europaea* L.) is one of the most extensive crops in the countries that bordered the Mediterranean Sea. Almost six million ha of olive trees are cultivated worldwide, from which the 98% of them are on the Mediterranean countries, especially on Italy, Greece, and Spain. As a matter of fact, one million ha of olive trees are only cultivated in Spain. Fruit and oil obtained from *Olea europaea* L. have been widely studied for its alimentary use, one of the main pillars of the MD (Figure 1). During the recollection of olive trees and the production of olive oil, huge quantities of olive by-products are generated with no practical applications, which have been studied to their application in cosmetic, pharmaceutical, and food industry. For instance, olive leaves represent the 10% of the weight of olives before processing. Furthermore, this part of the tree is the main site of plant metabolism, where primary and secondary plant products are produced from photosynthesis. For that, olive leaves can be considered potential sources of bioactive compounds, such as oleuropein or HXT.

### 2.1. Extra Virgin Olive Oil

The main source of HXT is extra virgin olive oil (EVOO), once of the principal ingredients of MD, that is used as cooking fat and salad dressing. EVOO is rich in unsaturated fatty acids (especially oleic) and phenolic groups, as antioxidant substances, followed by tocopherols and carotenes, that are also present [14]. The phenols detected in EVOO can be divided into alcohols, acids, flavonoids, lignans and psecoiridoids. In fact, HXT is the most important psecoiridoid in EVOO.

Great variations in the concentration of these antioxidant compounds exist according to one olive oil or another (0.02–600 mg/kg), which can occur due to factors such as the olive variety, ripening, processing or the region and cultivation technique used [15]. These compounds are characterized by their antimicrobial, antioxidant, anti-inflammatory, and anti-cancer biological properties. Numerous studies have demonstrated the capacity of EVOO phenolic groups to reduce the excess of free radicals that can cause oxidative damage [16,17,18,19,20,21].

### 2.2. Hydroxytyrosol

HXT (or 4-(2-dihydroxyphenyl) ethanol) is known to be one of the most powerful natural antioxidant extracts, and it is just below gallic acid [22]. In this sense, part of its bioactivity lies in the fact that the fourth carbon of its catechol ring (benezene-1,2-diol) have been substituted by a 2-hydroxyethyl group. Thus, HXT is ten times more antioxidant that green tea and two times more antioxidant than coenzyme Q10 (ubiquinone) and is also effective as anti-inflammatory and antineoplastic compound [22]. HXT is a phenylethanoid whose antioxidant properties have been proved in vitro; it is obtained from olive leaves; it is characterized by an intense flavor and aroma; and oleuropein is its precursor [23,24]. Moreover, the antioxidant capacity in vivo of HXT has been proved in several studies with rats, as in Merra et al. [25] or in Lemonakis et al. [26], who showed the power of HXT to reduce the risk of suffering from metabolic syndrome. In fact, in the chemical structure of HXT, the compound has an additional hydroxyl group in its benzene ring, compared to tyrosol (TYR). Therefore, it obtains a greater function as a free radical scavenging, which means an increase in its antioxidant power, as well as in its efficacy under stress conditions [26].

## 3. Health Benefits

As a matter of fact, several studies have shown that HXT, which is known to be a highly bioactive ortho-diphenol, has interesting antioxidant and antimicrobial characteristics and important beneficial effects (Table 1) on the cardiovascular system and on several human diseases [13,27,28,29]. The list of biological activities turns out to be inexhaustible, including a negative regulation of the immune response, which protects human erythrocytes from hydrogen-peroxide-induced oxidative damage, and anti-inflammatory, antithrombotic, and hypocholesterolemia activities. In addition, it is a powerful monoamine oxidase (MAO-B) inhibitor, which makes HXT a perfect compound for the treatment of Alzheimer’s, Parkinson’s, and other neurological diseases [27,28]. In addition, HXT has been proved to be a powerful superoxide anion and a hydroxyl radical scavenger. As a result, this compound protects cells located in many parts of the human anatomy from damage and death, which results in a decrease of cell death frequency and a significant prolongation in the mean of the cell life [13].

From our last review [22], research on the health benefits of the consumption of olive by-products have been growing until today (Table 1).

In this way, cardiovascular benefits have been widely demonstrated by several authors, as in vitro as in vivo, both in animals and humans. For instance, Carnevale et al. [30] showed as 10–20 g EVOO per day, following a Mediterranean Diet, reported a decrease in the production of oxidative markers in the post-prandial period in 25 middle-age subjects. After that, Violi et al. [31] also showed cardiovascular and anti-diabetic benefits in humans after following a Mediterranean Diet including 10 g EVOO per day. These benefits implied a reduction of blood glucose and the LDL cholesterol, which improved the insulin level while reducing the oxidized LDL in blood. These results agree with Carnevale et al. [33], who also showed the same behavior after the intake of 20 mg oleuropein extract per day. In relation to this effect, also in humans, Boronat et al. [40,41] reported an improve of the endothelial function by the consumption of 25 mg tyrosol extract per day. Furthermore, Davis et al. [32] showed lower systolic blood pressure after three and six months in >64 years-old humans (n = 137) who followed a Mediterranean Diet including 14.8 mL EVOO per day.

Moreover, this cardiovascular benefit was also observed in mice after an intake of 5 mg HXT/kg/day, which implied a reduction of the oxidative stress even in combination with a high-fat diet [36]. In addition, El-azem et al. [37] reported a decrease of the oxidized LDL in rats with the consumption of 30 mL EVOO (89.4% HXT), which was also related with the reduction of the size of the tumour in vitro (MCF-7 and MDA-MB-231 breast cancer cell lines).

In particular, cardiovascular protection has been also associated to the prevention of steatosis in mice after application of 20 mg HXT/kg/day [35]. In addition, 100 µg HXT/day in rats has shown to promote an anti-inflammatory status, which has been also linked with its anti-cancer [43] and its anti-sclerosis benefits [34]. Besides, the accumulation of HXT in the brain of rats, after an intake of 5 mg/kg/day has suggested its neuroprotective activity by the reduction of the oxidative stress and, hence the protection of neuronal cells [42].

Knowing the anti-bacterial and anti-fungal benefits of HXT [38] and that this compound can increase de bioavailability of Fe and Zn with a minimum degradation during the digestion [13] after its incorporation to a meat product, there are plenty of reasons for incorporating this extract into meat products to extend their shelf life while improving their nutritional properties.

## 4. Olive and Its Derivatives as Functional Ingredients in Meat Product Production

According to its antioxidant and antimicrobial activities, olive derivatives have widely studied as food preservatives, especially in meat products. This compound has shown its antioxidant capacity in meat products, especially in those rich in unsaturated fatty acids. This is the case of, for example, sausages and frankfurters made with nuts and EVOO and also enriched in HXT to avoid lipid and protein oxidation [44,45]. In addition, HXT is an antioxidant compound, which can link to certain minerals (gluconate Fe (II) in black olives) and catalyzes its oxidation. Then, it may be possible that HXT affects to availability of some trace minerals [24]. In this way, last obtained results about the effect of olive derivatives incorporation into manufactured meat products are shown in Table 2. Furthermore, this incorporation has followed two different ways: the endogenous way, throughout the animal feed, and the exogenous way, either by the meat formula (making process) or by bioactive packed.

By the endogenous way, Mattioli et al. [46] incorporated 10% olive leaves extract to rabbit feed, which did not influence to the physical-chemical properties of rabbit meat. In fact, meat from rabbits fed with enriched diets in olive leaves showed an improvement in the fatty acid profile by increasing the content of oleic acid and its derivatives [46]. Similarly, Papadomichelakis et al. [47] reported an important improvement of fatty acid profile of the chicken meat from broilers feed with 50 g/kg/day dried olive pulp for 42 days. Moreover, Jabalbarezi Hukerdi et al. [48] and Jabalbarezi Hukerdi et al. [49] improved the proportions of unsaturated fatty acids and the oxidative stability of Mahabadi male goat kids meat after supplementation of 75 and 150 g olive leaves/kg/day. Lastly, similar results has been also obtained by El Otmani et al. [50] after addition of 200 g olive cake (made with olive leaves)/kg/day to the diet of male goat kids.

Regarding the incorporation of olive derivatives by the bioactive packed, Moudache et al. [51] elaborated a multilayer polyethylene film with different doses (from 2 to 15%) of olive leaf extract. These films were used to pack fresh minced pork meat for 16 days at 4 °C and olive leaf extract incorporation enhanced the stability of fresh meat against lipid and protein oxidation processes. In a similar form, Bermúdez-Oria et al. [52] used bioactive edible films with 0.1 and 0.5% HXT and 3,4-dihydroxyphenylglycol extracts to preserve beef meat for 7 days at 4 °C. As a result, the edible film enriched in olive phenols and made of pectin and fish gelatin was effective to control the lipid oxidation of raw beef meat. As a matter of fact, it delayed lipid oxidation by 100% for 7 days, which can be justified by the combined effect of the film, as oxygen barrier, and the incorporation of HXT as antioxidant agent.

Nevertheless, the easiest way to preserve new “super foods” has been the exogenous way, by incorporating olive derivatives into meat formulas.

In this way, Robert et al. [53] reformulated low fat pork meat systems by incorporating 100 mg oleuropein/kg. As a result, pork meat reported a good physical stability during refrigerated storage for 14 days. In fact, the encapsulation of oleuropein hindered the degradation of the antioxidant compound (oleuropein), leading to meat with lower contents of peroxide and malondialdehyde, as well as higher antioxidant capacity. Furthermore, Zhu et al. [54] reduced the fat content by increasing the content of MUFA (especially oleic acid) while reducing SFA in Harbin dry sausages throughout the incorporation of 4% EVOO. In fact, the addition of healthy fat replacers provided the formation of muscle protein gels. Besides, the reduction of fat also decreased the lipid oxidation and the sensory perception of the sausages [54].

Particularly, our research group has recently developed different meat products exogenously enriched in HXT, as main antioxidant compound obtained from olive leaves. For instance, chicken nuggets with 750 ppm HXT from olive leaf presented a reduction of the microbial growth, a better oxidative stability, and a good sensory quality for 12 months at −18 °C [55]. In addition, in a pioneering way, we have compared the action in meat derivatives of this derivative of the olive tree, as a natural source of HXT, against HXT synthetically obtained.

## 5. New Trends: Natural vs. Synthetic Hydroxytyrosol

Olive oil extraction involves different processes such as extraction, olive washing, beating and grinding. A number of different byproducts are originated during olive oil production, such as leaves, wastewater, and pomace, and the uses of which can be the focus of a sustainable valorization in innovative products such as HXT [60]. According to Luque de Castro and Japón-Luján [61], leaves have the most potent radical scavenging power of the different parts of olive trees. Specifically, in olive leaves there are several a great diversity of compounds such as, substituted phenols (hydroxytyrosol, tyrosol, vanillin, caffeic acid, vanillic acid) flavones (diosmetin, apigenin-7-glucoside, diosmetin-7-glucoside, luteolin, and luteolin-7-glucoside), flavan-3-ols (catechin), flavonols (rutin), and secoiridoids (oleuropein).

Natural HXT is mainly found in olive leaves fruits, and waste waters from olive oil production, where it is naturally created through the hydrolysis of oleuropein. Natural HXT extracts from byproducts of olive oil industry contains a maximum of 7–25% of pure HXT, along with other bioactive olive compounds. The natural mechanism by means of which olive trees form free HXT is enzymatic hydrolysis, and it involves enzymes glucosidase and esterase [62]. As a matter of fact, procedures that generate high amounts of HXT by means of the use of by-products obtained after the milling and extraction of olive oil have been considered in several studies. The water waste from olive mills have been studies after the extraction, since such water is very rich in free HXT [44]. This is, indeed, the best way to use the large amounts of waste generated during the production of olive oil. However, knowing the broad health benefits of the HXT molecule, other industrial methods have been developed to obtain extracts with a higher purity degree. Alternatively, synthetic enzymatic HXT procedures have been suggested [63], but acid hydrolysis from oleuropein is the most used mechanism to obtain this antioxidant in industrial processes. It is in this way where HXT extracts can reach a purity level of 94–99%.

**Figure 2:**
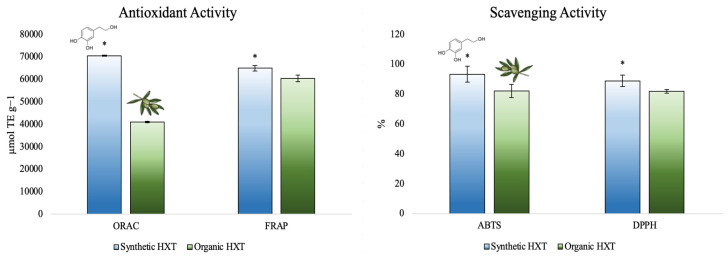
Total antioxidant capacity of synthetic and organic HXT [58]. * Denotes significant differences between synthetic and organic HXT.

For instance, our research group has evaluated the antioxidant capacity by several methods (oxygen radical absorbance capacity -ORAC-, and Ferric reducing antioxidant power -FRAP- methods) and the scavenging activity (against 2,2 -azino-bis(3-ethylbenzothiazoline-6-sulfonic acid)-ABTS- and 2,2-diphenyl-1-picrilhidrazilo -DPPH-) of the synthetic HXT following different ways and obtaining promised results (Figure 2).

As appreciated, after measuring the antioxidant capacity of both extracts through several methods, HXT obtained from the synthetic source reported the highest antioxidant and scavenging activity compared to natural HXT obtained from olive leaf, which is directly related to the purity of both extracts. This behavior is useful for the optimization and production of medicines and foods enriched in HXT to enhance their health benefits.

For that reason, we tested these extracts in two different meat products. Firstly, 200 ppm of HXT, from both origins, reduced the microbial growth, the protein and the lipid oxidation being the synthetic HXT the only that did not alter the sensory quality of fresh lamb burgers for 6 days at 4 °C [58]. In this manner, we tested these extracts also a dry-cured pork sausage (‘fuet’) obtaining similar results [59]. In this sense, although on these days society prefers natural products than synthetic, the stability of the natural isolated HXT may have lower and limited stability, which may reduce its bioactivity. For that reason, encapsulation of the natural product may avoid the negative flavor problems and increase its activity.

In this way, companies as the Chinese Shandong Bailong Chuandyuan BIO-TECH Co., Ltd., the German Willy Benecke GMBH, and the Spanish Coralim ADITIVOS S.L. are experts in organic additives markets. In the same way, there are more companies focused on development, production, and distribution of free additives solutions used by the meat industry, for example, Murcian Catalina Food Solutions S.L. (Spain). Other multinational industries also focused on production of natural flavorings, have also aimed their view in food industry, such as the Spanish Indukern Food Division, which has developed the Blend-a-Kern CFX, CEX, and CII solutions for the elaboration of manufactured meat products without E numbers, or the Israeli Frutarom Industries Ltd., which specializes in the production and distributions of natural extracts obtained from herbs, fruits, and vegetables for flavor and fragrances.

This kind of companies gives the possibility to the meat industry to elaborate clean label meat products. For example, North American Coleman Natural Foods Llc. is launching a new clean label meat products line, such as beef burgers, chicken sausages, and maple-smoked bacon. In addition, French ActiMeat^®^ is on the lookout for clean label and organic products with the aim objective of develop natural, authentic, and respectful with environment meat products. In the Spanish market, Domínguez Meat Products, SL. has developed the “Bo&San” line, which includes meat products free of additives and allergens, such as “Spanish cured “chorizo,” bacon, roast ham, Celtic cured ham, and burgers, while Mafriseu S.A. produces traditional manufactured meat products, such as sausages, meatballs, minced meat, Spanish “chorizo,” burgers, “butifarra,” paté, or “fuet,” among others. In addition, Noel Alimentaria S. L. sells since several years ago roast ham and roast poultry breast free of additives.

## 6. Conclusions

Beneficial effects of olive and HXT consumption have been extensively studied due to its antioxidant, antimicrobial, and anti-inflammatory power. For this reason, in last 20 years, researchers have focused on the reduction and the removal of preservatives and dyes by olive derivatives and HXT incorporation to achieve “clean label” meat products. Unfortunately, HXT from olive leaf cannot be directly incorporated to manufactured meat products, since its characteristic flavor has been palatably unaccepted, as it has been described above. Therefore, it can be concluded that this incorporation can be reached by synthetic sources of HXT, as an ingredient in their formula, throughout its application in new systems of packaging or by encapsulation, are valid to obtain its health benefits and antioxidant properties on meat. Consequently, a great opportunity exists for meat products processors to use natural antioxidants, such as HXT, to replace synthetic additives while maintaining product quality.

## Figures and Tables

**Table 1:** Health benefits of olive derivatives consumption as in vitro as in vivo.

Health Benefit	Form/Specie	Dose Used	Model	Main Effects	Reference
Cardiovascular	EVOO included in a Mediterranean Diet	10 or 20 g/day	In Vivo: humans (25 subjects; 36 years old)	Prevented the production of ROS in the post-prandial period	[30]
Anti-diabetic and cardiovascular	EVOO included in a Mediterranean Diet	10 g/day	In Vivo: humans (25 subjects)	Reduction of blood glucose, LDL cholesterol and its oxidized form, and improved insulin level in comparison with the use of corn oil	[31]
Cardiovascular	EVOO included in a Mediterranean Diet	14.8 mL/day	In Vivo: humans (137 subjects; >64 years old)	Lower systolic blood pressure and improved endothelial function	[32]
Anti-diabetic and cardiovascular	Oleuropein in a basal diet	20 mg/day	In Vivo: humans (20 subjects; 33 years old)	Reduces glucose and oxidative markers while increases insulin blood levels	[33]
Anti-sclerosis	EVOO and HXT in a basal diet	20 mg/kg/day	In Vivo: rats (25 subjects)	Reduces oxidation of lipids and proteins while increases levels of glutathione peroxidase.	[34])
Anti-diabetic benefit and Hepatic protection	HXT in a basal diet	20 mg/kg/day	In Vivo: mice (10 subjects)	Prevention of hepatic steatosis	[35]
Cardiovascular	HXT in a high-fat diet	5 mg/kg/day	In Vivo: mice (28 subjects)	Reduce cardiovascular risk by reduction of oxidative stress and increase of plasma antioxidant profile	[36]
Cardiovascular and anti-tumor	EVOO (89.4 HXT)	30 mL	In Vitro: breast cancer cell lines (MCF-7 and MDA-MB-231) In Vivo: rats (22 subjects)	Decrease the oxidation of LDL cholesterol. Improve of the endothelial function, which reduced the size of the tumour	[37]
Anti-bacterial and anti-fungal	HXT	100–500 mM	In Vitro: Standard fungal strains: A. *nidulans*, A. *fumigatus*, A. *flavus*, F. *oxysporum* and C. *albicans*. Standard bacterial strains: P. *aeruginosa*, E. *coli*, *Klebsiella* sp., P. *fluorescens*, S. *aureus,* and B. *subtilis.*	Strong antifungal activity in studied fungal strains. High efficiency in fungal plasma membrane destruction.	[38]
Cardiovascular and anti-diabetic	HXT	9.7 mg/day	In Vitro: hepatic cell lines (HepG2)	Improvement pancreatic β-cell responsiveness which produces an increase in insulin sensitivity	[39]
Cardiovascular	Tyrosol	25 mg/day	In Vivo: humans (33 subjects)	Improved endothelial function, increased HDL cholesterol and antithrombin IIII, while decreased plasma homocysteine, and gene expression in peripheral blood mononuclear cells.	[40,41]
Neuro-protector	HXT	5 mg/kg/day	In Vivo: rats (8 subjects)	Brain accumulation of HXT produced the reduction of the oxidative stress at neuronal level, which suggested its neuroprotective activity	[42]
Anti-inflammatory and anti-tumour	HXT	100 µg/day	In Vivo: rats (8 subjects)	Oleuropein acts as pro-inflammatory status, whereas HXT promotes an anti-inflammatory status.	[43]
Digestive	HXT	150 ppm	In Vitro: CACO-2 cell lines	Increase bioavailability of Fe and Zn	[13]

EVOO: extra virgin olive oil; ROS: reactive oxygen substance.

**Table 2:** Olive derivatives used as a preservative in meat products.

Incorporation	Extract Form	Dose Used	Meat Product	Storage Conditions	Main Effects	Reference
Packed (multilayer polyethylene film)	Olive leaves extract	2, 5, 10, and 15%	Fresh minced pork meat	16 days at 4 °C, packed under MAP conditions (30% CO_2_ and 70% O_2_)	Improved the stability of fresh minced meat against oxidation.	[51]
Packed (bioactive edible films)	HXT and 3,4-dihydroxyphenylglycol extracts	0.1 and 0.5%	Beef meat	6–7 days at 4 °C, packed under aerobic conditions	The combination of the film, which acted as oxygen barrier, and the HXT as antioxidant compound reduced lipid oxidation by 100% for 7 days.	[52]
Animal feed	Olive leaves extract	10%	Rabbit meat	-	It did not affect to meat characteristics, but fatty acid profile was improved by increase the oleic acid content.	[46]
Animal feed	Dried olive pulp	25 and 50 g/kg/day	Broiler chicken (42 days) meat	-	Meat was enriched in monounsaturated fatty acids, such as c18:1 n-9. Higher concentration rates (than 50 g/kg) may negatively affect to pH and color of breast meat.	[47]
Animal feed	Olive leaves extract	75 and 150 g/kg/day	Mahabadi male goat kids meat	-	Increased the content of MUFA (especially LA, LNA, and CLA), PUFA, PUFA:SFA ratio, and decreased hh n-6/n-3 ratio in lamb meat.	[49]
Animal feed	Olive leaves extract	75 and 150 g/kg/day	Mahabadi male goat kids meat	-	It did not affect to meat characteristics while increased the antioxidant properties of the meat by increasing the olive leave dose.	[48]
Animal feed	Olive cake (made with dry matter of olive leaves)	200 g/kg/day	Male goat kids meat	-	It did not affect to physical characteristics, fat, or meat quality.	[50]
Making process	HXT from olive leaf and from olive fruit extracts	200 ppm	Fish patties	11 days at 4 °C, packed under aerobic conditions	Reduction of the TVC and the lipid oxidation measured by nonanal, 1,6-octadien-3-ol, and hexanal presence after 11 days.	[56]
Making process into double emulsions as fat replacers	Olive leaves extract	100 mg oleuropein/kg	Pork meat systems (10.4% fat and 15% muscle)	14 days at 4 °C and 7 days at 60 °C to study the oxidative stability	High stability and antioxidant activity. Oleuropein encapsulation avoid its degradation, which kept its antioxidant properties against lipid oxidation.	[53]
Making process	HXT from vegetable water extract	200 ppm	Fish patties	14 days at 4 °C, packed under aerobic conditions	Reduction of the TVC, the lipid and the protein oxidation, and the trimethilamine content.	[57]
Making process	HXT from olive leaf extract	750 ppm	Chicken nuggets	12 months at −18 °C, packed under aerobic conditions	Reduction of the TVC, the lipid and the protein oxidation.	[55]
Making process	HXT from an organic source (olive leaf) and from a synthetic source extract	200 ppm	Lamb burgers	6 days at 4 °C, packed under aerobic conditions	Both extracts reduced the TVC, the protein oxidation and the lipid oxidation measured by nonanal and hexanal presence after 6 days. However, only the synthetic HXT did not alter the sensory quality of the lamb burgers.	[58]
Making process as fat replacer	Olive oil	4%	Low-fat Harbin dry sausages	36 days at room temperature	Reduced the fat content, increased the MUFA (especially oleic acid), reduced lipid oxidation, and improved sensory perception of sausages.	[54]
Making process	HXT from an organic source (olive leaf) and from a synthetic source extract	200 ppm	Dry-cured pork sausage: fuet	100 days at 5 °C	Both extracts reduced the TVC, the protein oxidation and the lipid oxidation. However, only the synthetic HXT did not alter the sensory quality of the fuet.	[59]

MAP: modified atmosphere packaging; TVC: total viable count; SFA: saturated fatty acids; MUFA: monounsaturated fatty acids; PUFA: polyunsaturated fatty acids; LA: linoleic acid; CLA: conjugated linoleic acid; LNA: linolenic acid.
